# A MATLAB-Based Boundary Data Simulator for Studying the Resistivity Reconstruction Using Neighbouring Current Pattern

**DOI:** 10.1155/2013/193578

**Published:** 2013-05-28

**Authors:** Tushar Kanti Bera, J. Nagaraju

**Affiliations:** Department of Instrumentation and Applied Physics, Indian Institute of Science Bangalore, Bangalore, Karnataka 560012, India

## Abstract

Phantoms are essentially required to generate boundary data for studying the inverse solver performance in electrical impedance tomography (EIT). A MATLAB-based boundary data simulator (BDS) is developed to generate accurate boundary data using neighbouring current pattern for assessing the EIT inverse solvers. Domain diameter, inhomogeneity number, inhomogeneity geometry (shape, size, and position), background conductivity, and inhomogeneity conductivity are all set as BDS input variables. Different sets of boundary data are generated by changing the input variables of the BDS, and resistivity images are reconstructed using electrical impedance tomography and diffuse optical tomography reconstruction software (EIDORS). Results show that the BDS generates accurate boundary data for different types of single or multiple objects which are efficient enough to reconstruct the resistivity images for assessing the inverse solver. It is noticed that for the BDS with 2048 elements, the boundary data for all inhomogeneities with a diameter larger than 13.3% of that of the phantom are accurate enough to reconstruct the resistivity images in EIDORS-2D. By comparing the reconstructed image with an original geometry made in BDS, it would be easier to study the inverse solver performance and the origin of the boundary data error can be identified.

## 1. Introduction

Electrical impedance tomography (EIT) [1, 2] reconstructs the spatial distribution of electrical conductivity or resistivity of a closed conducting domain (*Ω*) from the surface potentials developed by a constant current injection through the surface electrodes surrounding the domain to be imaged. Before carrying out the practical measurements on patients, it is advised to test an EIT system with a tissue mimicking model of known properties [3] called practical phantoms [4–10]. Hence, phantoms are often required to assess the performance of EIT systems for their validation, calibration, and comparison purposes. Two-dimensional (2D) EIT (2D-EIT) assumes that the electrical current flows in a 2D space which is actually three-dimensional inside real volume conductors. Hence, the development of a perfect 2D practical phantom is a great challenge as the real electrodes always have a definite surface area, and hence the injected current signal cannot be confined in a 2D plane in bathing solution [5]. Researchers have developed a number of practical phantoms which are three-dimensional objects, and those phantoms are designed and developed, generally, for their own EIT systems. Practical phantoms containing electrolyte (or other conducting medium) [4–10] are three-dimensional in shape and hence they will have some data error due to the three dimensional current conduction. Also, the phantoms containing electrolytes (e.g., NaCl solution or saline) [5, 7, 8] are difficult to transport and are prone to errors since the evaporation of the water gives rise to changes in conductivity [9]. In addition, temperature variations have a marked effect on the conductivity because the temperature coefficient is large [11]. Therefore, the practical phantoms will have a poor stability and a gradually increasing data error over time. Network or mesh phantoms [12, 13] are compact, more stable, rugged, portable, easy to move, consistent over time, and less temperature dependent. But these phantoms need a huge number of identical electronic components properly designed in a mesh mimicking the conductivity distribution of a practical biological tissue. Furthermore, for a large tissue structure, a mesh phantom requires a huge number of very precision components. The reproduction of these kinds of phantoms having different properties is often time-consuming [14]. The option for changing the position and property of an inhomogeneity is limited by the phantom structure and the number of elements in mesh phantom but the practical phantoms allow us to put several types of object in different positions in the bathing solution, but they produce several errors contributing to the poor signal to noise ratio (SNR) in boundary data.

Reconstructed image quality in impedance tomography depends on the errors associated with practical phantom, electronic hardware, and inverse solver performance. Image quality is largely affected by the practical phantom design parameters such as phantom geometry, electrode geometry, electrode materials, and the nature and behavior of the inhomogeneity and bathing solution. SNR is also reduced by the error contributed by current injector, data acquisition system, and signal conditioner circuits. In practical phantoms, the voltage data developed by a three-dimensional current conduction are collected form surface electrodes connected to an analog instrumentation. Therefore, it is quite confusing to identify the source of the errors responsible for poor image quality in a 2D-EIT system. In order to overcome the difficulties and limitations of practical and mesh phantoms, a MATLAB-based boundary data simulator (BDS) is developed to generate accurate 2D boundary data for assessing the EIT inverse solvers. BDS is an absolute 2D data simulator which is required to generate the errorless 2D boundary data to study and modify the inverse solver of a 2D EIT system. As the BDS is a computer program, it is free from the instrumentation errors and allows us to generate voltage profile with different types of phantom geometry, inhomogeneity and background conductivity profile, and inhomogeneity geometry (shape, size, and position). Moreover, it is absolutely stable, compact, easy to use, and easy to handle and modify for further development. Boundary data for different phantom geometries are generated in BDS, and resistivity images are reconstructed in standard reconstruction algorithm. BDS is studied to conform its suitability to use for boundary data generation with different phantom configurations which are required to assess the EIT inverse solvers. 

## 2. Methods

### 2.1. Mathematical Modelling of EIT

EIT image reconstruction is a nonlinear inverse problem [15] in which the electrical conductivity distribution of a closed domain (*Ω*) in a volume conductor is reconstructed from the surface potential data developed at the boundary (∂*Ω*) by injecting a constant current signal. A low frequency and low magnitude constant sinusoidal current is injected through an array of electrodes attached to the boundary, and the boundary potentials are measured using a data acquisition system. The voltage data collected from surface electrodes are then used by an image reconstruction algorithm [15] which reconstructs the conductivity distribution of the domain under test (DUT). The reconstruction algorithm computes the boundary potential for a known current injection and known conductivity values and tries to compute the conductivity distribution for which the difference between the measured boundary potential (*V*_*m*_) and the calculated (*V*_*c*_) is minimum. The reconstruction algorithm is developed with two parts: forward solver (FS) [5, 15–17] and inverse solver (IS) [15–17]. Forward solver calculates the boundary potential data for a known current injection and known conductivity values. Inverse solver computes the conductivity distribution for which the boundary voltage difference (Δ*V* = *V*_*m*_ − *V*_*c*_) becomes minimum.

The DUT will have the distinct conductivity values at each points defined by their corresponding coordinates (*x*, *y*). Due to a constant current injection, a potential profile is developed within DUT, and its potential profile without any internal energy sources depends on the conductivity profile. Hence, a relationship, called EIT governing equation, between the electrical conductivity (*σ*) of the points within the DUT and their corresponding potential values (Φ) can be established. The governing equation in EIT [1, 2] can be derived from the Maxwell's equation and can be represented as

(1) $$\begin{array}{c} \nabla \cdot \sigma \nabla \Phi =0. \end{array}$$

To calculate the domain potential developed for a constant current injected to the DUT with a known conductivity distribution, the above equation is essentially to be solved. As the EIT governing equation is a nonlinear partial differential equation, the direct or analytical technique fails to solve it. Therefore, to calculate the domain potential, the equation is solved by developing a mathematical model called “forward model” which is derived from (1) using a numerical technique like finite element method (FEM) [18]. 

The EIT governing equation has an infinite number of solutions, and hence the FEM formulation of the EIT technique is essentially required to be provided by some boundary conditions [18–20] to restrict its solutions space. The boundary conditions are imposed into the FEM formulation of EIT by specifying the value of certain parameters (voltage or current). The parameters defining the boundary conditions may be either the potentials at the surface or the current density crossing the boundary or mixed conditions.

The boundary conditions, in which the parameters are the potential at the surface, are called the *Dirichlet* boundary conditions and are represented as [1, 5, 19, 20]

(2a) $$\begin{array}{c} \Phi =\Phi_{i}, \end{array}$$

where *i* = 1,…, *N* are the measured potentials on the electrodes.

The boundary conditions, in which the parameters are current density crossing the boundary, are known as the *Neumann* boundary conditions [1, 5, 19, 20] which are given by 

(2b) $$\begin{array}{c} \int_{\partial \Omega }^{}\sigma \frac{\partial \Phi }{\partial n}=\{\begin{array}{cc} +I & \mathrm{on}\;\;\text{the}\;\;\text{source}\;\;\text{electrode}\\ -I & \mathrm{on}\;\;\text{the}\;\;\text{sink}\;\;\text{electrode}\\ 0 & \text{otherwise}, \end{array} \end{array}$$

where ∂*Ω* is the boundary, and *n* is the outward unit normal vector on an electrode surface.

In EIT, the FEM technique is used to derive the forward model from the governing equation in the form of a matrix equation establishing the relationship between the injected current and the developed potential within a DUT. The relationship can be assumed as the transfer function of the system which is mathematically represented as a matrix called global stiffness matrix (GSM) [18] or transformation matrix constructed with the elemental conductivities (*σ*) and nodal coordinates (*x*, *y*). In EIT, FEM discretizes the DUT by a finite element mesh containing finite number of elements of defined geometry and finite number of node. FEM applied on the governing equation to derive the forward model of a DUT in the form of a matrix equation using the *σ* and nodal coordinates. In the EIT forward model, the relationship established between the current injection matrix [*C*] (matrix of the applied signal) and the nodal potential matrix [Φ] (matrix of the developed signal) through the transformation matrix [*K*(*σ*)] is mathematically represented as

(3) $$\begin{array}{c} \left[\Phi \right]={\left[K\left(\sigma \right)\right]}^{-1}\left[C\right]. \end{array}$$

Now, in FEM formulation in EIT, when the current matrices [*C*] and [*K*(*σ*)] are known, and the nodal potential matrix [Φ] is unknown, the forward model or the mathematical problem is termed as the “*forward problem*”. The procedure of calculating the [Φ] by solving the forward problem (3) with known [*K*(*σ*)] and known [*C*] is termed as “*forward solution*”. In EIT, the forward solver first computes the potential distribution with the assumed initial conductivity distribution (*σ*_0_) with a known constant current simulation, and then the inverse solver reconstructs the conductivity distribution from the measured boundary potential data for a same constant current injection through surface electrodes. The EIT reconstruction algorithm tries to mathematically find the elemental conductivity values (conductivity distribution) for which the difference between the estimated nodal potentials (*V*_*c*_) computed in the FS and the potentials measured (*V*_*m*_) on the surface electrodes (for a same current injection values) becomes minimum.

The inverse solver of the EIT reconstruction algorithm is developed with a mathematical minimization algorithm (MMA) [19–22] such as Gauss-Newton-based mathematical minimization algorithm (GN-MMA). In GN-MMA, the conductivity update vector ([Δ*σ*]) is calculated and the boundary data mismatch vector (Δ*V*  =  *V*_*m*_ − *V*_*c*_) is minimized by an iteration technique like the modified Newton-Raphson iteration technique (NRIT) [19–22]. The [Δ*σ*] matrix is the desired variation in the elemental conductivity values in [*σ*] matrix for which the forward solver calculates the boundary potentials more similar to the measured value in next iteration using NRIT. Therefore, the algorithm starts with an initial elemental conductivity vector ([*σ*_0_]), and it is then updated to ([*σ*_1_] = [*σ*_0_] + [Δ*σ*]) in the next iteration. Using this [*σ*_1_], FS calculates a new potential distribution in DUT and a new voltage mismatch vector [Δ*V*_1_] is thus obtained and compared with the previous voltage mismatch vector [Δ*V*_0_]. If the Δ*V*_1_ is not found as the minimum, the iteration process is continued till the *k*th iteration using the conductivity update vector ([Δ*σ*_*k*_]) developed by GN-MMA. Using, NRIT the [*σ*] matrix is iteratively updated to [*σ*_*k*+1_] = [*σ*_*k*_]+[Δ*σ*_*k*_] and repetitively tries to find out the minimum value of [Δ*V*].

Hence, in the EIT inverse solver, it is understood that the desired elemental conductivity matrix is obtained by a minimization algorithm (MMA) which is composed of Gauss-Newton method and Newton-Raphson iteration in which the technique iteratively tries to find out an optimum conductivity distribution [*σ*_*k*_] for which the voltage mismatch vector is minimized [Δ*V*]. At a particular iteration in this MMA, the elemental conductivity matrix is calculated when the current matrices [*C*] and [Φ] or [Δ*V* = *V*_*m*_ −  *V*_*c*_] are known. This process is logically an opposite process to the forward problem. Thus, when the current matrices [*C*] and [Φ] are known, and the elemental conductivity matrix [*σ*] is unknown, the model or the problem is called the “*inverse problem*.” The procedure of calculating the [*σ*] or [Δ*σ*] using with known [Δ*V*] and the known [*C*] is termed as “*inverse solution*.”

### 2.2. Image Reconstruction with GN-MMA and NRIT

Electrical conductivity imaging is a highly nonlinear and ill-posed inverse problem [19–22]. In EIT, a minimization algorithm is used to obtain an optimized elemental conductivity value [*σ*] for which the voltage mismatch vector [Δ*V*] becomes minimum. In the image reconstruction process, the minimization algorithm [17, 18] first defines an objective function (*s*) from the computational predicted data [*V*_*c*_] and the experimental measured data [*V*_*m*_] and runs iteratively to minimize it. Generally, in the EIT image reconstruction algorithm, the inverse solver searches for a least square solution of the minimized object the function (*s*) using by a Gauss-Newton method and the NRIT-based iterative approximation techniques. 

If *f* is a function mapping a *t*-dimensional (*t* is the number of element in the FEM mesh) impedance distribution into a set of *M* (number of the experimental measurement data ([*V*_*m*_]) available) approximate measured voltages, then the Gauss-Newton-method-based minimization algorithm [19–26] tries to find a least square solution of the minimized object function (s) [19–26]  which is defined as:

(4) $$\begin{array}{c} s=\frac{1}{2}{||V_{m}-f||}^{2}=\frac{1}{2}{\left(V_{m}-f\right)}^{T}\left(V_{m}-f\right). \end{array}$$

Now, differentiating (4) with respect to the conductivity *σ*, it reduces to

(5) $$\begin{array}{c} s^{\prime }=-{\left[f^{\prime }\right]}^{T}\left[V_{m}-f\right]=-J^{T}\Delta V, \end{array}$$

where the matrix *J* = *f*′ is known as Jacobin matrix [19–22], which may be calculated by a method as described in [19, 22] or by the adjoint method [23] represented by (6)

(6) $$\begin{array}{c} J=\oint_{\Omega }^{}\nabla \Phi_{s}\cdot \nabla \Phi_{d}d\Omega , \end{array}$$

where Φ_*s*_ is the forward solution for a particular source location, and Φ_*d*_ is the forward solution for the adjoint source location (source at the detector location and detector at the source location).

Differentiating (5) with respect to *σ* again, the equation reduces to

(7) $$\begin{array}{c} s^{\prime \prime }={\left[f^{\prime }\right]}^{T}\left[f^{\prime }\right]-{\left[f^{\prime \prime }\right]}^{T}\left[V_{m}-f\right]. \end{array}$$

By Gauss-Newton method, the conductivity update vector [Δ*σ*] is given by

(8) $$\begin{array}{c} \Delta \sigma =-\frac{s^{\prime }}{s^{\prime \prime }}=\frac{J^{T}\Delta V}{{\left[f^{\prime }\right]}^{T}\left[f^{\prime }\right]-{\left[f^{\prime \prime }\right]}^{T}\Delta V}. \end{array}$$

Thus, the conductivity update vector is given by

(9) $$\begin{array}{c} \Delta \sigma ={\left[{\left[f^{\prime }\right]}^{T}\left[f^{\prime }\right]-{\left[H\right]}^{T}\Delta V\right]}^{-1}J^{T}\Delta V, \end{array}$$

where the higher-order term *H* = [*f*′′] is known as the *Hessian matrix *[24]. In (9) by neglecting *H*, the update conductivity vector reduces to

(10) $$\begin{array}{c} \Delta \sigma ={\left[{\left[f^{\prime }\right]}^{T}\left[f^{\prime }\right]\right]}^{-1}J^{T}\left[\nabla V\right]. \end{array}$$

In general, using NRIT method, the conductivity update vector expressed as in (10) can be represented for *k*th iteration (where *k* is a positive integer) as

(11) $$\begin{array}{c} \Delta \sigma_{k}={\left[{\left[J_{k}\right]}^{T}\left[J_{k}\right]\right]}^{-1}{\left[J_{k}\right]}^{T}\left[\Delta V_{k}\right], \end{array}$$

where [Δ*V*_*k*_] and [*J*_*k*_] are the voltage mismatch matrix and Jacobian matrix, respectively.

The [*f*′]^*T*^matrix in (11) is always ill conditioned [19–24], and hence small measurement errors will make the solution of (11) changes greatly. In order to make the system well posed, the regularization method [19–26] is incorporated into the reconstruction algorithm by redefining the object function [19–26] with regularization parameters as

(12) $$\begin{array}{c} s_{r}=\frac{1}{2}{||V_{m}-f||}^{2}+\frac{1}{2}\lambda {||G\sigma ||}^{2}, \end{array}$$

where *s*_*r*_ is the constrained least-square error of the regularized reconstructions, *G* is the regularization operator, and *λ* (the positive scalar) is called the regularization coefficient [19–26]

(13) $$\begin{array}{c} s_{r}=\frac{1}{2}{\left(V_{m}-f\right)}^{T}\left(V_{m}-f\right)+\frac{1}{2}\lambda {\left(G\sigma \right)}^{T}\left(G\sigma \right). \end{array}$$

Differentiating the inject function in (12) with respect to the elemental conductivity: the following relations are obtained

(14) $$\begin{array}{c} s_{r}^{\prime }=-{\left(f^{\prime }\right)}^{T}\left(V_{m}-f\right)+\lambda {\left(G\right)}^{T}\left(G\sigma \right), \end{array}$$

(15) $$\begin{array}{c} s_{r}^{\prime \prime }={\left(f^{\prime }\right)}^{T}\left(f^{\prime }\right)-{\left(f^{\prime \prime }\right)}^{T}\left(V_{m}-f\right)+\lambda G^{T}G. \end{array}$$

Now, using Gauss-Newton- (GN-) method-based minimization process, the conductivity update vector [Δ*σ*] is obtained as

(16) $$\begin{array}{c} \Delta \sigma =\frac{s_{r}^{\prime }}{s_{r}^{\prime \prime }}=\frac{{\left(f^{\prime }\right)}^{T}\left(V_{m}-f\right)-\lambda {\left(G\right)}^{T}\left(G\sigma \right)}{{\left(f^{\prime }\right)}^{T}\left(f^{\prime }\right)-{\left(f^{\prime \prime }\right)}^{T}\left(V_{m}-f\right)+\lambda G^{T}G}. \end{array}$$

Neglecting the *Hessian matrix *[24] in (15)

(17) $$\begin{array}{c} \Delta \sigma =\frac{s_{r}^{\prime }}{s_{r}^{\prime \prime }}=\frac{{\left(f^{\prime }\right)}^{T}\left(V_{m}-f\right)-\lambda {\left(G\right)}^{T}\left(G\sigma \right)}{{\left(f^{\prime }\right)}^{T}\left(f^{\prime }\right)+\lambda G^{T}G}. \end{array}$$

Replacing *f*′ by *J* and *G* ^*T*^ *G* by  *I* (identity matrix) (21) reduces to

(18) $$\begin{array}{c} \Delta \sigma =\frac{J^{T}\left(V_{m}-f\right)-\lambda I\sigma }{J^{T}J+\lambda I}, \end{array}$$

where the matrix *J* = *f*′ is the Jacobin as stated earlier.

Thus, the conductivity update vector ([Δ*σ*]) is found as

(19) $$\begin{array}{c} \Delta \sigma ={\left(J^{T}J+\lambda I\right)}^{-1}\left(J^{T}\left(V_{m}-f\right)-\lambda I\sigma \right). \end{array}$$

Sometimes, the last term (*λIσ*) is neglected [22], and the conductivity update vector [Δ*σ*] is calculated as

(20) $$\begin{array}{c} \Delta \sigma ={\left(J^{T}J+\lambda I\right)}^{-1}J^{T}\left(V_{m}-f\right). \end{array}$$

In general, the EIT image reconstruction algorithm provides a solution of the conductivity distribution of the DUT for the *k*th iteration as

(21) $$\begin{array}{c} \sigma_{k+1}=\sigma_{k}+{\left({\left(J^{T}J+\lambda I\right)}^{-1}\left(J^{T}\left(V_{m}-f\right)-\lambda I\sigma \right)\right)}_{k}. \end{array}$$

The EIT algorithm starts with the solution of FP obtained from the EIT governing equation, and the [*V*_*c*_] is calculated for a known current injection matrix [*C*] and an initial guess (known or assumed) conductivity matrix [*σ*_0_]. The voltage mismatch matrix [Δ*V*] is estimated, and then it is used to calculate the conductivity update matrix [Δ*σ*] using GN-MMA and is added to the initial conductivity matrix ([*σ*_*o*_]) to update it to a new conductivity matrix [*σ*_1_ = *σ*_*o*_ + Δ*σ*] using NRIT. New update matrix [*σ*_1_] is used in forward solver to obtain a new calculated boundary data matrix [*V*_*c*1_] which provides a new voltage mismatch matrix [Δ*V*_1_]. Therefore, the NRIT algorithms iteratively calculate the [Δ*σ*] using GN-MMA to find out an optimized [*σ*] matrix for which the [Δ*V*] reaches its minimum value. Thus, the EIT reconstruction algorithm is found to work in the following sequences:

(1) forward solver calculates the boundary potential matrix [*V*_*c*_] for a known current injection matrix [*C*] and an initial guess (known) conductivity matrix [*σ*_0_],
(2) measured voltage data matrix  [*V*_*m*_] is compared with [*V*_*c*_] to estimate the [Δ*V*] as [Δ*V* = *V*_*c*_ − *V*_*m*_],
(3) Jacobian (*J*) is computed,
(4) conductivity update vector [Δ*σ*] is calculated by Gauss-Newton-based minimization algorithm,
(5) [*σ*_*o*_] matrix is updated to a new conductivity matrix [*σ*_1_ = *σ*_*o*_ + Δ*σ*] by adding [Δ*σ*] to [*σ*] using Newton-Raphson iteration technique (NRIT),
(6) new update matrix [*σ*_1_] is used in forward solver to calculate the new voltage mismatch matrix [Δ*V*_1_],
(7) check whether the [Δ*V*_1_] is minimum or not or compare the [Δ*V*] with a specified error limit (*ε*) if provided,
(8) stop the algorithm if Δ*V* ≤ *ε* condition is achieved, otherwise repeat the steps 1 to 7 until the specified stopping criteria (Δ*V* ≤ *ε*) is achieved.

### 2.3. Boundary Data Simulator (BDS)

A two-dimensional boundary data simulator (BDS) is developed in MATLAB R2010a [27] using finite element method (FEM) [15] to generate accurate boundary data for studying the EIT reconstruction algorithms. The MATLAB-based BDS is developed as an absolute 2D data simulator for EIT image reconstruction studies, and it is used suitably to generate the errorless 2D boundary data to study and modify the inverse solver of a 2D EIT system. As BDS is developed in a computer software, it is found free from errors produced by the EIT instrumentation and phantom. BDS also allows us to generate boundary potential data for different type of phantom geometry, inhomogeneity geometry (shape, size, and position), inhomogeneity conductivity profiles, and background conductivity profiles. Moreover, it is developed as a compact, absolutely stable, and easy to use and handle for EIT studies. It is developed in such a way that it can be modified for further modifications.

BDS is developed with MATLAB-based computer program consisting of four-part imaging domain simulator (IDS), EIT model developer (EMD), current injection simulator (CIS), and boundary data calculator (BDC). Imaging domain simulator (IDS) in BDS simulates a domain with inhomogeneity with their corresponding conductivity distributions. EIT model developer (EMD) derives a mathematical model of the forward solver by applying FEM on the governing equation of the DUT in the form of a matrix equation. Current injection simulator (CIS) simulates a constant current injection through the definite points at the domain boundary with neighbouring current injection protocol [1, 2, 28–30]. The boundary data calculator (BDC) solves the governing equation by solving the forward model and calculates the potentials at all electrodes at the domain boundary.

**Figure 1:**
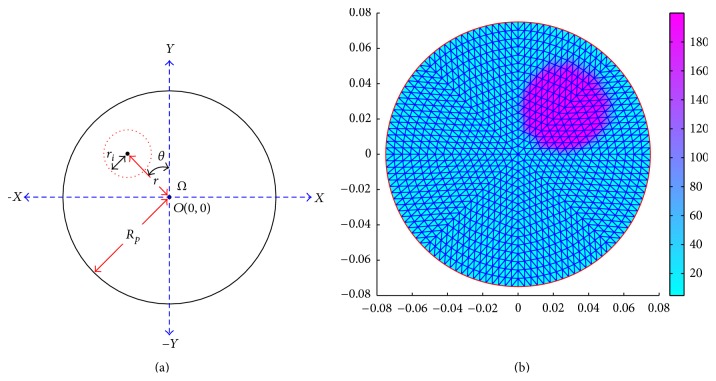
(a) A circular phantom domain (Ω) with an inhomogeneity defined by polar coordinate (*r* and *θ*); (b) a phantom domain (discretized by an FE mesh with 2048 elements and 1089 nodes) with a circular inhomogeneity (*R*_*p*_ = 75 mm, *r*_*i*_ = 25 mm, *r* = 37.5 mm,   *θ* = 45°,  *σ*_*i*_ = 0.005 S/m, and *σ*_*b*_ = 0.21 S/m).

Imaging domain simulator (IDS) first defines a DUT with a desired area (*A*_*D*_) defined by a required diameter and defined with a particular coordinate system. Imaging domain simulator applies the FEM to discretize the domain with a 2D finite element mesh containing finite element of triangular elements (*t*) and finite number of nodes (*n*). In IDS, a circular domain (Ω) to be imaged is defined with a required radius (*R*_*p*_) using the Cartesian coordinate system (Figure 1(a)), and the domain is discretized with a finite element (FE) mesh (Figure 1(b)). The mesh is symmetrically composed of the first-order triangular elements with linear shape functions [18, 31]. The FE mesh is generated with the *pdetool* of MATLAB R2010a in such a way that it can be refined further to increase the number of elements as per the requirement. All the coordinates and parameters assigned to the finite elements and the nodes are stored in corresponding matrices. Boundary nodes are identified, and the sixteen nodes among the boundary nodes are assigned as the electrodes called the electrode nodes. Inside the domain one (or more) smaller region (regions) is (are) defined as the inhomogeneity (inhomogeneities) positioned at a particular place. The center point (*P*) of the inhomogeneity with the required shape and size is positioned inside the phantom domain by defining its center with a polar coordinate (*r*, *θ*) as shown in Figure 1(a). Single or multiple inhomogeneities are defined with their desired areas (*A*_*I*_) inside the DUT, and elements within the inhomogeneity and the background are identified. The background area is defined as the area of the domain surrounding the inhomogeneity (*A*_*B*_ = *A*_*D*_ − *A*_*I*_), and the elements within the background area (*A*_*B*_) are identified. The elements within the inhomogeneity are assigned with a particular conductivity called inhomogeneity conductivity, (*σ*_*i*_) while the rest of the elements are assigned with a different conductivity called background conductivity (*σ*_*b*_) as shown in Figure 1(b). The assigned conductivity values of all the elements are assumed to be featured at their corresponding centroids. 

EIT model developer (EMD) develops the mathematical model of the forward solver by applying FEM on the governing equation and derive the forward model of a DUT in the form of a matrix equation (3) using the elemental conductivities and nodal coordinates. The EMD establishes a relationship between the current injection matrix, [*C*] (matrix of the applied signal), and the nodal potential matrix, [Φ] (matrix of the developed signal), through the transformation matrix [*K*(*σ*)] which is mathematically represented by (3). The global stiffness matrix [*K*(*σ*)] in EIT is actually an admittance matrix [23] that is formed [16] using the nodal coordinates of all the elements with their corresponding conductivities. Thus, the [*K*(*σ*)] inforward model represents the transfer function of the EIT system obtained from the governing equation by FEM formulation [19].

The current injection simulator (CIS) is used to simulate a constant current injection through the sixteen nodes called simulated electrodes (SE) on the domain boundary with neighbouring current injection protocol. The CIS works in a “*for*” loop to execute all the projections [1, 28, 30, 32] of current injection process. In BDS, a constant current injection is simulated into the DUT surrounded by the sixteen simulated current electrodes (SE_*I*_) with all the possible combination of SE_I_ pairs, and the potential data are calculated on all the electrodes called voltage electrodes (SE_*V*_) in BDC. The current injection through a particular current electrode pair (say SE_*I*1_ and SE_*I*2_) and corresponding voltage data collection from all the possible voltage electrodes (SE_*V*1_, SE_*V*2_, SE_*V*3_, SE_*V*4_, SE_*V*15_, SE_*V*16_, SE_*V*7_, SE_*V*8_, SE_*V*9_, SE_*V*10_, SE_*V*11_, SE_*V*12_, SE_*V*13_, SE_*V*14_, SE_*V*15_ and SE_*V*16_) is known as a simulated current projection (SCP). Hence, in an *N*-electrode EIT system, there will be *N*-different current projections each of which will inject current through a particular current electrode pair and collect *m* voltage (differential/grounded) data where *m* may be either equal to *N* or less than *N* depending on the EIT data collection strategy called the current pattern [1, 28, 30, 32]. Therefore, a complete scan (containing all the current projections) conducted on the DUT yields *N* × *m* voltage data. As the BDS is studied for sixteen electrode system, the CIS runs for sixteen times and provides sixteen current projections (SCP_*V*1_, SCP_*V*2_, SCP_*V*3_, SCP_*V*4_, SCP_*V*15_, SCP_*V*16_, SCP_*V*7_, SCP_*V*8_, SCP_*V*9_, SCP_*V*10_, SCP_*V*11_, SCP_*V*12_, SCP_*V*13_, SCP_*V*14_, SCP_*V*15_, and SCP_*V*16_). Therefore, a complete data collection procedure (called a complete scan) in the BDS collects *m* voltage data from the voltage electrodes or voltage electrode pairs in all the sixteen current projections and computes 16 × *m* voltage data.

Boundary data calculator (BDC) calculates the potentials (developed for a constant current injection by CIS) at all electrode points (electrode nodes) at the domain boundary in each current projection for a particular current pattern. The current injection matrix [32] is formed in CIS using the *Neumann type boundary conditions, *and the potential matrix is calculated from (3) using the matrix inversion technique working on *L-U* factorization [33] process. The BDS is developed to run in an another “*for*” loop for *m* times to calculate the *m* electrode potentials from voltage electrodes or voltage electrode pairs at each of the steps of the loop. This second “*for*” loop runs within the first “*for*” loop for *m* times and collects *m* voltage data for each step of first “*for*” loop and hence collects 16 × *m* voltage data as first “*for*” loop runs for sixteen times. Moreover, as the EIT reconstruction process needs a complete scan, the BDS runs in each current projection and computes sixteen electrode potentials at each projection. The domain potential is calculated from the forward model (3), and the potential values of all the nodes are stored in a nodal potential matrix [33, 34] denoted by [*M*_NP_]. Boundary potential data are separated from [*M*_NP_] and stored in a different matrix called boundary potential matrix [*M*_BP_]. The electrode potential data are extracted from the nodal potential matrix [*M*_NP_] and are stored in a separate matrix called electrode potential matrix [*M*_*EP*_]. In sixteen electrode EIT system, the [*M*_*EP*_] is formed as a column matrix and contains the 16 × *m* electrode potentials (differential or grounded) obtained for all the projections. 

### 2.4. Neighbouring or Adjacent Current Injection Method

**Figure 2:**
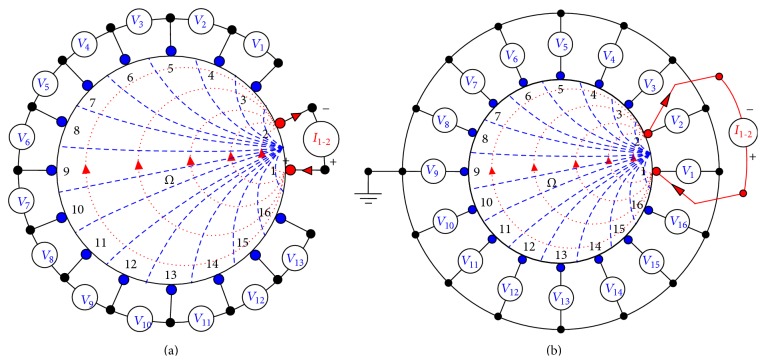
Current injection and boundary data collection in neighbouring current injection method; (a) data collection method as suggested by Brown and Segar and (b) data collection strategy as suggested by Cheng et al.

In neighbouring or adjacent current injection method, first reported by Brown and Segar [35], the current is applied through two neighbouring or adjacent electrodes, and the differential voltages is measured successively from all other adjacent electrode pairs excluding the pairs containing one or both of the current electrodes. For a sixteen electrode EIT system with domain under test surrounded by equally spaced sixteen electrodes (E_1_, E_2_, E_3_, E_4_, E_5_, E_6_, E_7_, E_8_, E_9_, E_10_, E_11_, E_12_, E_13_, E_14_, E_15_, and E_16_), the neighbouring method injects current through the current electrode pairs for sixteen current projections (Figure 2), and the differential voltages are measured across the voltage electrode pairs using four electrode method in each projection.

As shown in Figure 2(a) in the first current projection (P_1_) of adjacent method, the current is injected through electrode 1 (E_1_) and electrode 2 (E_2_), and the thirteen differential voltage data (*V*_1_, *V*_2_, *V*_3_,…, *V*_13_) are measured successively between the thirteen electrode pairs E_3_-E_4_, E_4_-E_5_,…, and E_15_-E_16_, respectively (Figure 2(a)). As reported by Brown and Segar, in neighbouring current injection method, the current density within the DUT is found highest between the current electrodes (E_1_ and E_2_ for P_1_); the current density then decreases rapidly as a function of distance [35]. Similarly, in current projection 2 (P_2_), the current signal is injected through electrodes 2 (E_2_) and 3 (E_3_), and an another set of thirteen differential voltage data (*V*_1_, *V*_2_, *V*_3_,…, *V*_13_) are collected between the thirteen electrode pairs E_4_-E_5_, E_5_-E_6_,…, E_16_-E_1_, and so on. Lastly, in the current projection 16 (P_16_), the last set of thirteen differential voltage data (*V*_1_, *V*_2_, *V*_3_,…, *V*_13_) are collected between the thirteen-electrode pairs E_2_-E_3_, E_3_-E_4_,…, and E_14_-E_15_ by injecting the current through the electrodes E_16_ and E_1_. Thus, the neighbouring current injection method in a sixteen electrode EIT system data collection procedure consists of sixteen current projections (P_1_, P_2_, P_3_,…, P_15_, and P_16_), and each of the current projection yields thirteen differential voltage data (*V*_1_, *V*_2_, *V*_3_, …, *V*_13_). Therefore, a complete data collection scan with the neighbouring current injection method in a sixteen electrode EIT system yields 16 × 13 = 208 voltage measurements.

Though in neighbouring method, EIT boundary data are not collected across the electrode pairs containing one or two current electrode for contact impedance problem [35], but sometimes it is advantageous to collect the boundary data from all the electrodes including the current electrodes to obtain the greatest sensitivity to the resistivity changes in the domain as reported by Cheng et al. [36]. In the present study, the boundary potentials are calculated at all the electrodes (Figure 2(b)) with respect to a virtual ground point selected within the DUT. Hence, in a complete data collection scan, the potentials on all the electrodes are collected in all the sixteen current projection and are stored in [*M*_*EP*_]. Therefore, the [*M*_*EP*_] is found as a column matrix containing 16 × 16 voltage data all collected with respect to the virtual ground point of the DUT. Hence, in the present study, with neighbouring current injection method, the [*M*_*EP*_] is found as a 256 × 1 matrix containing 256 electrode potentials. In the present study, 1 mA current injection is simulated through the electrodes of the simulated domain containing sixteen nodal electrodes using adjacent or neighboring current injection protocol (Figure 2(b)). The potentials on all the sixteen electrodes are calculated using boundary data calculator (BDC) for all the current projections, and the electrode potential matrix [*M*_*EP*_] is used as the calculate boundary potential matrix [*V*_*c*_] to reconstruct the conductivity distribution of DUT.

The BDS is designed in such a way that a huge number of voltage data sets can be generated using different types of phantoms with their different design parameters. Boundary potential data [*V*_*c*_] are generated for different type of phantom configurations, and the boundary data have been tested with electrical impedance tomography and diffuse optical tomography reconstruction software (EIDORS) [37, 38] for 2D-EIT. A large number of data sets are generated by changing the values of one or more phantom parameters like: phantom diameter (*D* = 2*R*_*p*_), inhomogeneity radius (*r*_*i*_), inhomogeneity geometry (shape, size, and position), inhomogeneity number (*N*_*i*_), bathing solution conductivity (*σ*_*b*_), and inhomogeneity conductivity (*σ*_*i*_). 1 mA current injection is simulated to the domain boundary, and corresponding boundary data sets are used for image reconstruction in EIDORS. Data generation in BDS and image reconstruction in EIDORS are studied for different inhomogeneity geometries in DUT. Reconstruction is also studied for different iterations and for multiple inhomogeneity reconstruction to evaluate the BDS. 

## 3. Results and Discussion

**Figure 3:**
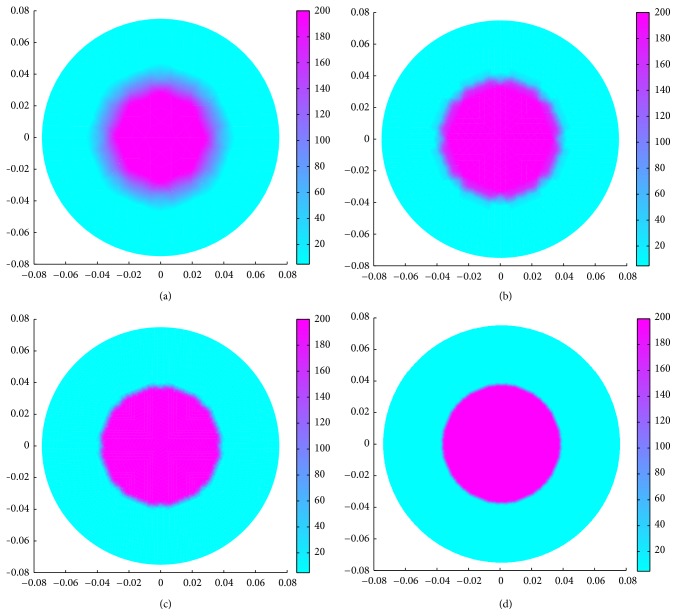
Circular inhomogeneity (*R*_*p*_ = 75 mm, *r* = 0,   *r*_*i*_ = 37.5 mm,   *σ*_*i*_ = 0.005 S/m,   and  *σ*_*b*_ = 0.21 S/m) with FEM mesh with different number of finite elements: (a) 512 elements and 289 nodes, (b) 2048 elements and 1089 nodes, (c) 8192 elements and 4225 nodes, and (d) 32768 elements and 16641 nodes.

Image reconstruction quality in EIT depends on the boundary data accuracy which is dependent on the geometric accuracy of the inhomogeneity developed in BDS. Dimensional accuracy of the inhomogeneity depends on the number of finite elements in the FE mesh or mesh refinement number (*N*_mr_) as shown in Figure 3. As the *N*_mr_ increases, the number of elements in the FE mesh is increased, and hence the geometric accuracy of the inhomogeneity increases which gives more accurate boundary data and better image reconstruction (Figure 3). But the BDS with a highly refined mesh needs a high PC memory and large computation time. In this paper, the mesh refinement is found suitable as *N*_mr_ = 4 as per the configuration of the PC (2.4 GHz/1.5 GBRAM/ P-IV) used. It is observed that the FE mesh with *N*_mr_ = 4 (containing 2048 elements and 1089 nodes) gives almost an accurate geometry (Figure 3) to the desired inhomogeneity and generates a reconstructible data set in less than 10 seconds. EIDORS reconstructs the resistivity images from the BDS data sets using regularized image reconstruction technique. 

**Figure 4:**
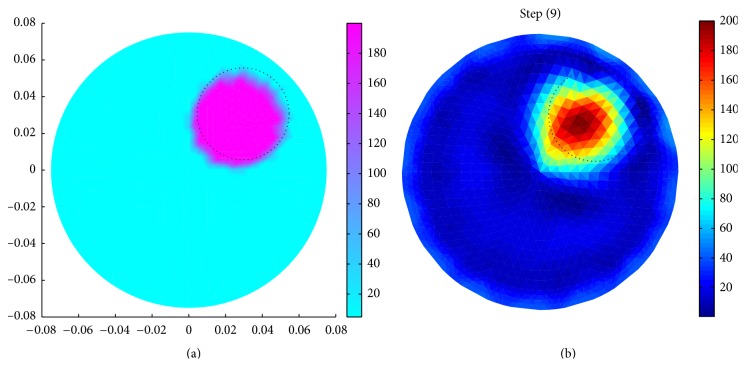
(a) Simulated domain with a circular object (*R*_*p*_ = 75 mm, *r* = 37.5, *θ* = 45°, *r*_*i*_ = 25 mm, *σ*_*i*_ = 0.005 S/m, and *σ*_*b*_ = 0.21 S/m); (b) reconstructed image of (a).

Results show that the resistivity or conductivity can be successfully reconstructed from the boundary data generated by our BDS using a circular domain (*R*_*p*_ = 75 mm) with a circular inhomogeneity (*r* = 37.5 mm, *r*_*i*_ = 25 mm, *θ* = 45°, *σ*_*i*_ = 0.005 S/m, and *σ*_*b*_ = 0.21 S/m) in the 9th iteration (Figure 4). It is also observed that the reconstructed shape of the inhomogeneity is similar to that of the original one (Figure 4(a)), and the reconstructed conductivity profile in Figure 4(b) is almost similar to that of the original object in Figure 4(a). 

**Figure 5:**
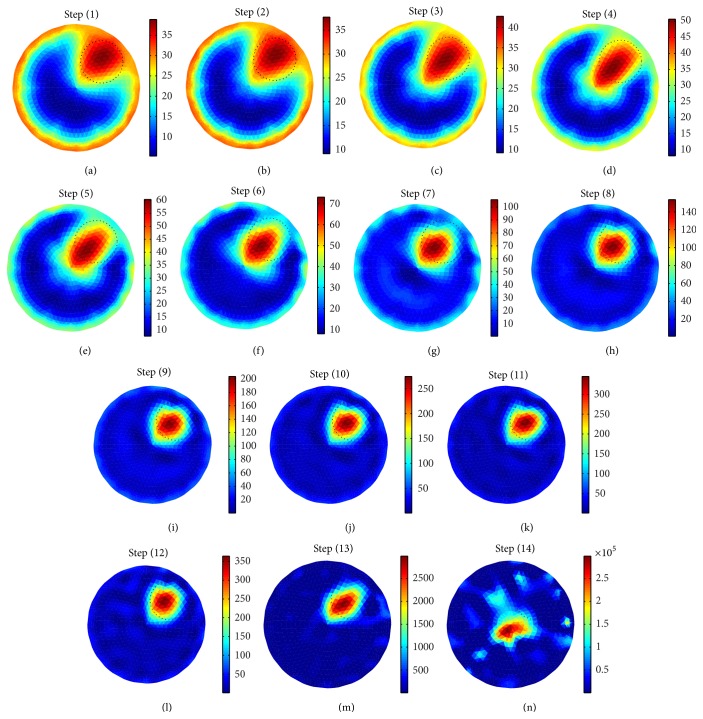
Reconstructed images of a simulated domain with a circular inhomogeneity (*R*_*p*_ = 75 mm, *r* = 37.5, *θ* = 45°, *r*_*i*_ = 25 mm, *σ*_*i*_ = 0.005 S/m, and *σ*_*b*_ = 0.21 S/m) for different number of iterations in inverse solver in EIDORS-2D: (a) 1st iteration, (b) 2nd iteration, (c) 3rd iteration, (d) 4th iteration, (e) 5th iteration, (f) 6th iteration, (g) 7th iteration, (h) 8th iteration, (i) 9th iteration, (j) 10th iteration, (k) 11th iteration, (l) 12th iteration, (m) 13th iteration, and (n) 14th iteration.

Iteration studies shows that in different reconstruction steps called iterations (Figure 5), the reconstructed images become more localized from iteration to iteration and the reconstruction errors (appeared by the red color at phantom periphery) are gradually reduced (Figure 5). 

It is observed that the resistivity is successfully reconstructed from the boundary data in the 9th iteration (Figures 5(i) and 5(j)), though the shape of all the reconstructed images in 9th–12th iterations is almost similar to that of the original one (shown by dotted circles in Figure 5). As the reconstructed resistivity profile similar to that of the original is obtained only in the 9th iteration, the 9th iteration is taken as the optimum reconstruction. In 13th and 14th iterations, the resistivity is overestimated, and the images are lost. The optimum iteration number depends on the data accuracy and reconstruction algorithm, and hence the BDS can be used to generate the boundary data sets required for assessing the inverse solver in EIT.

**Figure 6:**
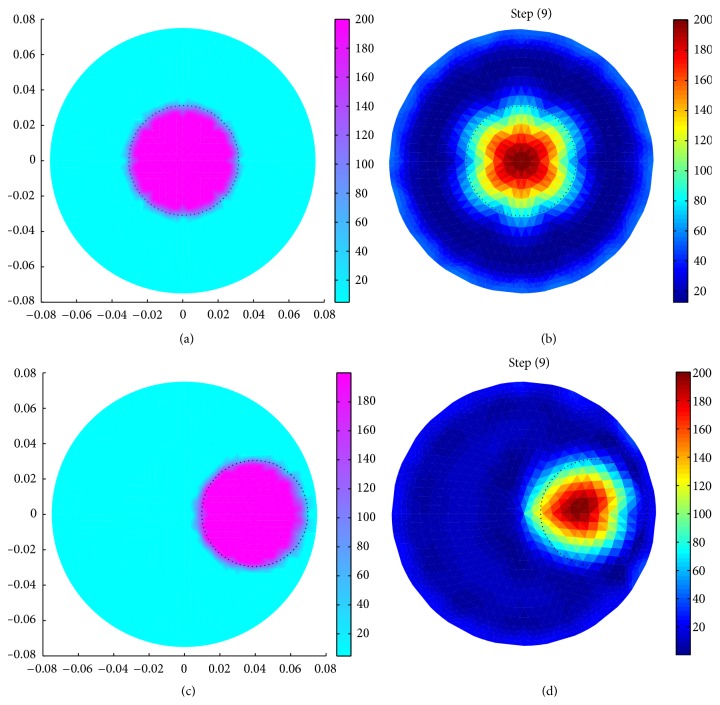
Image reconstruction of a simulated domain with a circular inhomogeneity (*R*_*p*_ = 75 mm, *r*_*i*_ = 30 mm, *σ*_*i*_ = 0.005 S/m, and *σ*_*b*_ = 0.21 S/m) at different positions (*r*, *θ*): (a) simulated domain with inhomogeneity at *r* = 0 mm and *θ* = 0°, (b) reconstructed image of the domain shown in Figures (a) and (c) simulated domain with inhomogeneity at *r* = 37.5 mm and *θ* = 0°, and (d) reconstructed image of the domain shown in Figure (c).

Voltage data are also generated for a domain (*R*_*p*_ = 75 mm) with the circular inhomogeneities (*r*_*i*_ = 25 mm, *σ*_*i*_ = 0.005,  S/m, and *σ*_*b*_ = 0.21 S/m) positioned at different places using the BDS (Figure 6). It is observed that the reconstructed image is more circular for an inhomogeneity positioned at the phantom centre where *r* = 0 and *θ* = 0° (Figure 6(a)). On the other hand, for *r* ≠ 0, that is, for the inhomogeneities near domain boundary (Figure 6(b)), reconstructed images are not perfectly circular because of the comparatively less accurate shape of the original object obtained for *r* ≠ 0. For a less number of mesh refinements, the geometry of the original side objects is not exactly circular itself (Figure 4), and hence the corresponding boundary data have lower accuracy. An FE mesh with large *N*_mr_ can easily produce an accurate geometry for the boundary objects (objects near domain boundary) with proper shape, which gives a boundary data without geometric error and automatically improves the image shape.

**Figure 7:**
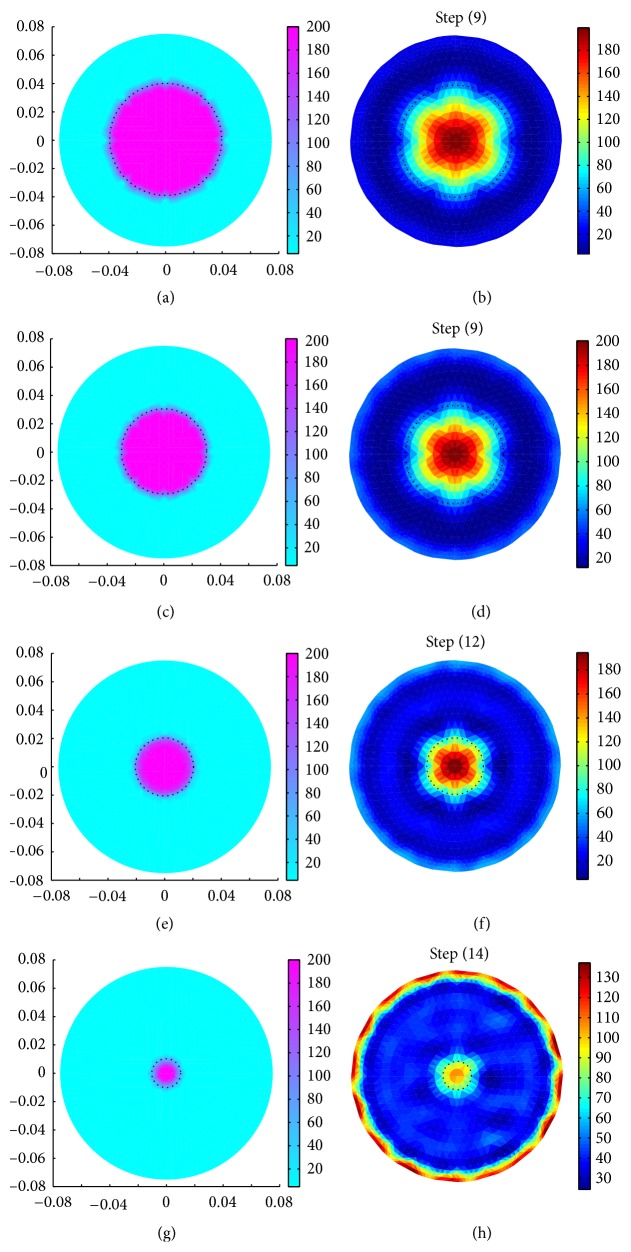
Image reconstruction of circular inhomogeneities (*R*_*p*_ = 75 mm, *r* = 0, *σ*_*i*_ = 0.005 S/m, and *σ*_*b*_ = 0.21 S/m) with different diameters: (a) original object with *r*_*i*_ = 40 mm, (b) reconstructed image of the object shown in (a), (c) original object with *r*_*i*_ = 30 mm, (d) reconstructed image of the object shown in (c), (e) original object with *r*_*i*_ = 20 mm, (f) reconstructed image of the object shown in (e), (g) original object with *r*_*i*_ = 10 mm, and (h) reconstructed image of the object shown in (g).

Boundary data sets are also generated with a circular domain (*R*_*p*_ = 75 mm and *σ*_*b*_ = 0.21 S/m) with a circular inhomogeneity (*σ*_*i*_ = 0.005 S/m) with different diameters (2*r*_*i*_) and all positioned at the phantom center (*r* = 0). The boundary data are calculated and used for reconstructing the resistivity images. Results show that for the domain discretized with *N*_mr_ = 4, the data sets, generated with a diameter larger than 13.3% of the phantom diameter, are accurate enough (Figures 7(a)–7(f)) to reconstruct the resistivity images in EIDORS-2D. It is clearly observed that for *N*_mr_ = 4, the triangular elements within the inhomogeneity with smaller *r*_*i*_ are unable to shape themselves into a proper circle (Figure 7(g)). Hence, the data obtained for the inhomogeneity with a diameter of 20 mm has low accuracy (Figure 7(g)), and hence the resistivity image (Figure 7(h)) is found with low resolution showed and some reconstruction error (appeared in the red color at phantom periphery). Increasing the FE elements in BDS, the boundary data error can be minimized, and the improved resistivity image can be achieved even for smaller inhomogeneities with a diameter less than 13.3% of *R*_*p*_.

**Figure 8:**
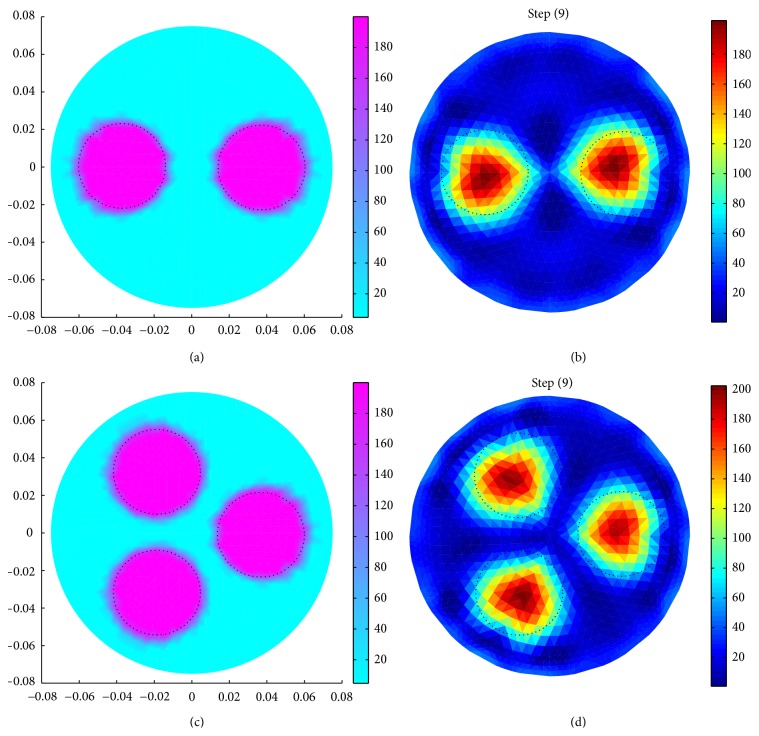
Image reconstruction of multiple inhomogeneities (*σ*_*b*_ = 0.21 S/m and *σ*_*i*_ = 0.005 S/m): (a) simulated domain with two circular objects (*r*_*i*_ = 25 mm, 180° apart), (b) reconstructed image of the domain shown in (a), (c) simulated domain with three circular objects (*r*_*i*_ = 25 mm, 120° apart), and (d) reconstructed image of the domain shown in (c).

Boundary potential data are also generated for domains (*R*_*p*_ = 75 mm) containing multiple circular inhomogeneities (*r*_*i*_ = 25 mm, *r* = 37.5 mm, *σ*_*i*_ = 0.005 S/m, and *σ*_*b*_ = 0.21 S/m) placed at different positions inside the domain (Figure 8).  Figure 8(a) shows a domain with two circular inhomogeneities (180° apart from each other) which are placed at a central distance (*r*) of 37.5 mm. Similarly, another domain with three circular inhomogeneities (120° apart from each other) placed inside the phantom domain is shown in Figure 8(c). All the inhomogeneities in both the domains are positioned at a central distance (*r*) of 37.5 mm. 1 mA current is simulated with the neighbouring current pattern, and the boundary data are collected for resistivity reconstruction. It is noticed that the resistivity images (Figures 8(b) and 8(d)) of inhomogeneities in both the domains are reconstructed successfully.

Results show that the boundary data simulator can be efficiently used to generate boundary potential data for a huge number of phantom configurations in less than 10 seconds. BDS is software-based virtual EIT phantom, and hence it has a number of advantages over the practical and mesh phantoms. The literatures [39–41] presenting the phantom simulations are limited, and they only discuss the software phantoms developed for their own systems. BDS is a software-based versatile boundary data simulator which generates boundary data suitable for studying the reconstruction algorithm required for several EIT systems, and hence it is better suited for assessing the performance of the inverse solver of 2D electrical impedance tomography.

## 4. Conclusions

A MATLAB boundary data simulator (BDS) is developed for studying the resistivity reconstruction in inverse solvers of 2D-EIT. BDS is developed with four parts: imaging domain simulator (IDS), EIT model developer (EMD), current injection simulator (CIS), and boundary data calculator (BDC). Imaging domain simulator (IDS) simulates a domain with single or multiple inhomogeneities of different geometries defined with their corresponding conductivity distributions, whereas the EIT model developer (EMD) derives a forward model using FEM to solve the governing equation of the DUT. Current injection simulator (CIS) simulates a constant current injection through the simulated electrodes positioned at the domain boundary with the neighbouring current injection protocol. The boundary data calculator (BDC) solves the forward model to solve the governing equation and calculates the potentials at all the simulated electrodes. Boundary data are generated with different type of domains simulated in BDS by changing its input parameters. Resistivity images are reconstructed from the boundary data using standard EIT reconstruction software called EIDORS, and the BDS is evaluated. It is observed that the BDS with FE mesh with 2048 elements can simulate an inhomogeneity of desired geometry with suitable accuracy. The BDS with 2048 elements suitably generates the boundary data for simulated domains containing the objects with different geometries which are found efficient for image reconstruction in EIDORS. Results also show that the conductivity or resistivity profiles of the domains simulated in BDS are successfully reconstructed from their corresponding boundary data generated for different type of single and multiple inhomogeneities. By changing the inhomogeneity position, diameter, and number in BDS, boundary data are successfully generated as well as the resistivity images are reconstructed successfully. Multiple inhomogeneity imaging shows that the BDS suitably generates boundary data with the desired accuracy, and the boundary data are found efficient for resistivity reconstruction in EIDORS. Results also show that for the simulated domains discretized with *N*_mr_ = 4, the boundary data sets generated for circular inhomogeneity with a diameter larger than 13.3% of the phantom diameter are accurate enough to reconstruct the resistivity images in EIDORS. Increasing the FE elements in BDS, the boundary data error can further be minimized, and the improved resistivity image reconstruction can be obtained even for smaller inhomogeneities. Hence, it is concluded that the BDS generated a number of boundary data sets which can suitably be used for inverse solver assessment in EIT.
